# Inflammation-Associated Microbiota Composition Across Domestic Animals

**DOI:** 10.3389/fgene.2021.649599

**Published:** 2021-06-21

**Authors:** Seika Hashimoto-Hill, Theresa Alenghat

**Affiliations:** Division of Immunobiology and Center for Inflammation and Tolerance, Cincinnati Children’s Hospital Medical Center and Department of Pediatrics, University of Cincinnati College of Medicine, Cincinnati, OH, United States

**Keywords:** microbiota, IBD, canine, feline, equine, bovine, porcine

## Abstract

Domestic animals represent important resources for understanding shared mechanisms underlying complex natural diseases that arise due to both genetic and environmental factors. Intestinal inflammation, particularly inflammatory bowel disease (IBD), is a significant health challenge in humans and domestic animals. While the etiology of IBD is multifactorial, imbalance of symbiotic gut microbiota has been hypothesized to play a central role in disease pathophysiology. Advances in genomic sequencing and analytical pipelines have enabled researchers to decipher the composition of the intestinal microbiota during health and in the context of naturally occurring diseases. This review compiles microbiome genomic data across domestic species and highlights a common occurrence of gut microbiome dysbiosis during idiopathic intestinal inflammation in multiple species, including dogs, cats, horses, cows, and pigs. Current microbiome data obtained from animals with intestinal inflammation are mostly limited to taxonomical analyses in association with broad clinical phenotype. In general, a pathogen or pathosymbiont were not detected. Rather, functional potential of the altered microbiota has been suggested to be one of the key etiologic factors. Among the domestic species studied, canine analyses are currently the most advanced with incorporation of functional profiling of microbiota. Canine IBD parallels features of the disease in humans, thus canines represent a strong natural model for human IBD. While deeper analyses of metagenomic data, coupled with host molecular analyses are needed, comparative studies across domestic species can reveal shared microbial alterations and regulatory mechanisms that will improve our understanding of intestinal inflammation in both animals and humans.

## Introduction

The mammalian intestine houses trillions of commensal microbes, collectively termed the microbiota. Host-microbiota symbiosis is supported by mounting studies with human subjects and lab animal models (Clemente et al., 2012). The importance of microbiota in the health of domestic animals has also been recognized for decades, yet largely supported by anecdotal evidence. In recent years, however, increasing studies on microbiota composition of companion and livestock animals using 16S based sequencing platforms have been conducted. In addition to advancing knowledge related to animal health, these studies help decipher pathophysiology of human diseases, as domestic animals naturally develop similar conditions. Microbiota findings across domestic species are summarized and compared below.

## Dual Roles of Microbiota

Microbiota support mammalian health by promoting multiple physiologic processes, including nutrient metabolism, immune regulation, intestinal development, and antimicrobial protection (Jandhyala et al., 2015). On the other hand, alterations in the composition and function of the microbiota is implicated in many disorders such as inflammatory bowel disease (IBD) (Schirmer et al., 2019). IBD includes Crohn’s disease and ulcerative colitis and affects millions worldwide (Ng et al., 2017). The etiology of IBD is multifactorial including genetics, epigenetics, and environmental factors. Although IBD has been associated with alterations in the composition of microbiota in patients (Gevers et al., 2014; Schirmer et al., 2018; Lloyd-Price et al., 2019), the causative impact of altered microbiota is still under investigation.

Most studies aimed at understanding the underlying nature of IBD have relied heavily on experimental or genetic murine-models of intestinal inflammation in which altered microbiota composition also frequently occurs (Gkouskou et al., 2014). However, comparative studies in other mammalian species with intestinal inflammation is likely to yield critical clues into shared microbiome-based etiologies that occur in natural disease. Gastrointestinal disease is one of the leading health issues of domestic animals. IBD and other idiopathic enteropathies in animals pose challenges due to a lack of understanding of the cause. Microbiota disturbance has long been suggested, but more recent microbiome research has been undertaken to directly test this hypothesis in animal/veterinary science. In this review, we discuss these studies and highlight evidence supporting that gut dysbiosis and intestinal inflammation in domestic mammals are tightly linked, similar to observations in humans and murine models.

## Gut Microbiota of Domestic Mammals

**FIGURE 1:**
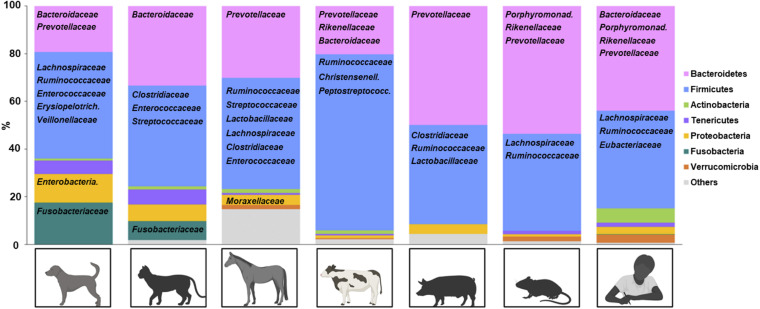
Representative fecal microbiota of healthy domestic animals. Phyla are represented by color and bacteria (family level) are represented below the corresponding phylum. Created with Biorender.com.

Dominance of two bacterial phyla, *Firmicutes* and *Bacteroidetes*, is unique to the mammalian hind gut ecosystem and not detected in fish intestine and plants (Mahowald et al., 2009; Ikeda-Ohtsubo et al., 2018). Microbiota of domestic animals generally follow this rule, with the exception that *Fusobacteria* can be one of the dominant phyla in canine and feline microbiota (Figure 1; Handl et al., 2011; Guard et al., 2015; Panasevich et al., 2015; Pilla and Suchodolski, 2020). Some symbiotic mechanisms provided by *Firmicutes* and *Bacteroidetes* include breaking down of dietary fibers and glycan to produce nutrients for colonocytes (Mahowald et al., 2009). Also, many indigenous *Firmicutes* and *Bacteroidetes* species are resistant to local antimicrobial peptides (Cullen et al., 2015). At lower taxonomic levels, microbiota compositions are distinct for each domestic species (Figure 1), possibly reflecting environment or host factors (Ley et al., 2008; Woo and Alenghat, 2017; Rothschild et al., 2018). Current knowledge of flora in domestic species during heath and inflammation are discussed below, focusing on data from 16S rRNA gene sequencing.

## Canine

Domestication of dogs is estimated to have occurred 12,000 years ago through acquisition of wild gray wolves (Larson and Fuller, 2014). Dogs have since shared environmental factors with humans. In particular, sharing food with human companions has impacted the microbiota composition of the dogs during evolution (Axelsson et al., 2013; Wang et al., 2013; Alessandri et al., 2019). Although large inter-individual variations were observed, five major phyla generally compose of dog microbiota including *Firmicutes, Bacteroidetes, Fusobacteria, Actinobacteria* and *Proteobacteria*. At the genus level, commonly isolated taxa in healthy dogs include *Clostridium, Eubacterium, Lactobacillus, Faecalibacterium, Ruminococcus, Prevotella, Fusobacterium, Bacteroides, Lachnospira*, and *Megamonas* (Handl et al., 2011; Guard et al., 2015; Panasevich et al., 2015; Pilla and Suchodolski, 2020).

Inflammatory bowel disease is a common non-infectious cause of chronic diarrhea and vomiting in dogs, with overrepresentation of certain breeds including German Shepherd, Norwegian Lundehund, Yorkshire terrier, Boxer and French bulldog (Dandrieux and Mansfield, 2019). While breed predisposition supports a genetic component, multiple studies have also shown association with dysbiosis in dogs. Some common microbiota findings were decreased *Firmicutes*, including *Clostridia* and *Faecalibacterium*, and decreased *Fusobacterium*, increase in *Gammaproteobacteria* including *Escherichia*. Alpha-diversity, which represents number of taxonomic groups, was decreased, and beta-diversity, which represents community similarity, was significantly different in IBD-microbiota compared to microbiota from non-IBD dogs (Table 1; Honneffer et al., 2014; Guard et al., 2015; Minamoto et al., 2015; Vázquez-Baeza et al., 2016; Niina et al., 2019). Aside from decreased *Fusobacterium* in canine IBD, these findings are similar to some human IBD studies (Pittayanon et al., 2020).

**TABLE 1:** Altered microbiota composition and function in intestinal inflammation across domestic animals.

Species	Taxa and functional categories decreased in inflammation	Taxa and functional categories increased in inflammation	Alpha-diversity	References
Dog	*Fusobacteria, Faecalibacterium, Bacteroidaceae. Prevotellaceae Clostridia* (IBD)	*Gammaproteobacteria. Escherichia* (IBD) Adherent-Invasive *E. coli* (Granulomatous colitis)	↓ in IBD, diarrhea	[Bibr B54][Bibr B24]; [Bibr B78][Bibr B57][Bibr B29]
	Amino acid metabolism (IBD) Methane metabolism, Oxidative phosphorylation, Folding catalysts (diarrhea)	Secretion system, Transcription factor (IBD) Benzoate degradation, Lipid metabolism. Flagellar assembly, Bacterial chemotaxis (diarrhea)		

Cat	*Roseburia, Megamonas, Helicobacter* (diarrhea) Unclassified *Clostridiales, Oscillospira, Ruminococcaceae* (IBD)	*Streptococcus, Enterobacteriaceae* (diarrhea, IBD) *Collinsella* (diarrhea), *Oscillospira* (IBD)	↓, in diarrhea. Not different from control in IBD	[Bibr B74][Bibr B51]
	RNA degradation, Biosynthesis of secondary metabolites, Biotin metabolism (diarrhea)	Phosphotransferase system, Transcription factors, Epithelial cell signaling, Lysine degradation, Tryptophan metabolism, Glycerolipid metabolism, Biodegradation of xenobiotics, Caprolactam degradation, Dioxin degradation, Benzoate degradation (diarrhea)		

Horse	*Actinobacteria, Spirochetes, Clostridiales* (Heliobactehaceae, Lachnospiraceae, Eubacteriaceae, Peptococcaceae, Clostridiaceae, Ruminococcaceae) (colitis)	*Fusobacteria (F. necrophorum, F. nucleatum, F. equinum)* (colitis)	Not different from control (colitis, diarrhea)	[Bibr B14][Bibr B4]

Cow	*Ruminococcaceae, Flavobacteriaceae, Desulfohalobiaceae*, Thermodesulfovibrionaceae, Odoribacteraceae, *Veillonellaceae* (diarrhea)	*Clostridia, Lachnospiraceae, Corynebacteriaceae*, (diarrhea)	↓ in diarrhea	Zeineldin et al., 2018
		Alanine, aspartate, and glutamate metabolism, Translation, Chromosome replication/repair, (diarrhea)		
	Carbohydrate metabolism, Pyruvate metabolism, Arginine and proline metabolism (diarrhea)			

Pig	*Bacteroides, Ruminococcaceae, Oscillospira, Christensenellaceae, Clostridiales, Prevotella, Lachnospiraceae* (diarrhea)	*Enterococcus, Trueperella, Enterobacteriaceae, Fusobacterium, Clostridium* (diarrhea)	↓ prior to onset of diarrhea in post-weaning pig	Dou et al., 2017
	mRNA surveillance pathway, Steroid biosynthesis (diarrhea)	Biosynthesis of type II polyketide products (diarrhea)		

Human	*Anaerostipes, Atopobium, Bifidobacterium, Christensenellaceae, Coriobacteriaceae, Lachnospira, Methanobrevibacter* (CD) *Akkermansia, Coprococcus*, *Eubacterium rectale, Lentisphaerae, Mollicutes* (UC) *Faecalibacterium prausnitzii* (CD/UC)	*Actinomyces, Escherichia, Veillonella* (CD) *Fusobacteria* (UC)	↓ or not different from control in CD	Pittayanon et al., 2020
		Oxidative stress, Auxotrophy, Benzoate metabolic pathway (CD) Magnesium-importing ATPase, Ethanolamine ammonia-lyase, Glutathione-disulfide reductase (CD/UC)		
	Basic biosynthesis (CD) Pyruvate synthase, Precorrin-2 dehydrogenase (CD/UC)			

*IBD, Inflammatory Bowel Disease; CD, Crohn’s Disease; UC, Ulcerative colitis.*

In contrast, a recent meta-analysis study comparing canine and human IBD metagenomes found distinct dysbiosis networks in canine versus human IBD microbiota (Vázquez-Baeza et al., 2016). Further studies are required to elucidate whether certain taxonomic groups found in multispecies have causative roles in IBD, or whether the imbalance of microbiota has collective functional effects. So far, a limited number of canine studies have conducted functional analyses of microbiota in the context of intestinal inflammation (Table 1; Guard et al., 2015; Minamoto et al., 2015). Interestingly, enrichment of the benzoate metabolism pathway was identified in the microbiota of dogs with diarrhea and this pathway has also been described as enriched in microbiota from IBD patients (Gevers et al., 2014; Guard et al., 2015; Lewis et al., 2015; Tataru and David, 2020).

One interesting exception in canine IBD is granulomatous colitis (GC) of Boxer and French bulldogs, in which the causative agent has been clearly associated with adherent-invasive *E. coli* (AIEC) (Simpson et al., 2006). Eradication of AIEC with antibiotics cures this disease (Manchester et al., 2013). Susceptibility to AIEC invasion was linked to polymorphism in SLAM family members/CD48 gene in the recent canine GWAS study (Hayward et al., 2016). Because AIEC has been isolated from subsets of human Crohn’s disease (CD) patients, and granulomatous lesions in canine GC mimic some CD lesions, microbiological comparisons between dog and human AIEC may elucidate a subset of CD pathophysiology (Dogan et al., 2020). Some canine studies have demonstrated beneficial effects of fecal microbiota transfer in IBD (Makielski et al., 2019; Niina et al., 2019), but factors that lead to improvement still need to be identified (Cammarota and Ianiro, 2019). Overall, canine microbiome analyses are the most advanced among domestic species and canine IBD may serve as a strong natural model for human IBD.

## Feline

Unlike dogs that classify as omnivorous, cats are obligate carnivores and may not rely much on microbiome fermentation for energy. Nevertheless, commensal microbes inhabit the feline intestine at similar levels to those in the dog and human (Johnston et al., 2001; Sender et al., 2016; Pilla and Suchodolski, 2020), and bacterial fermentation occurs in the feline intestine (Whittemore et al., 2019; Lyu et al., 2020). Similar to dogs, the major bacterial phyla in the domestic cat intestine are *Firmicutes, Bacteroidetes, Fusobacteria, Actinobacteria* and *Proteobacteria* (Figure 1; Ramadan et al., 2014; Suchodolski et al., 2015; Lyu et al., 2020). Lower taxa commonly found include *Clostridium, Enterococcus, Streptococcus, Bacteroide, Faecalibacterium, Fusobacterium*, and *Collinsella* (Minamoto et al., 2012; Rochus et al., 2014; Lyu et al., 2020).

Idiopathic intestinal inflammation resulting in diarrhea is a common occurrence in domestic cats and differences in microbiota composition have been observed in cats with intestinal inflammation (Table 1; Suchodolski et al., 2015; Marsilio et al., 2019). For example, *Enterobacteriaceae* and *Streptococcus* were increased in affected animals in two studies. Alpha-diversity was decreased when feline patients exhibited diarrhea, but this parameter was not significantly different in IBD in the second study. Functional analysis of microbiota from the cats with diarrhea demonstrated enrichment of the following pathways: phosphotransferase system, tryptophan metabolism, dioxin degradation and xylene degradation (Table 1; Suchodolski et al., 2015). Interestingly, the benzoate degradation pathway found in dogs and humans also trended upwards in feline samples (adjusted *p*-value 0.07). In addition, microbiota composition has been associated with other feline conditions such as obesity and diabetes (Kieler et al., 2016, 2019). Although only a few studies have been conducted for cates, feline dysbiosis has been demonstrated with intestinal inflammation.

## Equine

The horse is a monogastric herbivore that depends heavily on hind gut fermentation for its energy and nutritional requirements. *Firmicutes*, the major phylum fermenting dietary fibers in the equine gut, generally exhibits the largest relative abundance, followed by *Bacteroidetes*. Presence of *Actinobacteria, Tenericutes, Proteobacteria, Verrucomicrobia, Fibrobacteres*, and *Spirochetes* are also commonly reported in the equine intestine, albeit with much smaller abundances. Representative taxa at the genus level in healthy horses include *Clostridium, Ruminococcus*, *Streptococcus, Butyrivibrio* (Family *Lachnospiraceae), Fibrobacter, Lactobacillus, Prevotella*, and *Enterococcus* (Figure 1; Costa et al., 2012; Kauter et al., 2019; Stewart et al., 2019; Arnold et al., 2020). Balanced host-microbiota symbiosis in horses not only provides nutrition, but is also needed for healthy intestinal homeostasis. This is exemplified by the condition of antibiotic-induced equine colitis in which only a few doses of antibiotics can trigger fatal, non-infectious enterocolitis in horses (Cohen and Woods, 1999).

Inflammatory bowel disease in horses is less commonly reported partly due to difficulty obtaining biopsies for definitive diagnosis. Microbiota studies are also not extensively described specifically in equine IBD (Boshuizen et al., 2018). Instead, microbiota analyses in equine gut have been more commonly studied in idiopathic colitis, which can be caused by antibiotics, or stress-triggering factors such as transportation and dietary changes. Two studies found an increase in *Fusobacteria* in horses with idiopathic colitis, and a decrease in *Clostridia* (Table 1; Costa et al., 2012; Arroyo et al., 2020). However, alpha-diversity was not significantly different between healthy horses versus horses with colitis in these studies. Instead, antibiotics predictably reduced diversity of microbiota in asymptomatic horses (Costa et al., 2015; Arnold et al., 2020). It is likely that the microbiota shifts during disease are dynamic in the horse intestine. Among *Fusobacteria* species increased in equine colitis, *Fusobacterium nucleatum* has also been associated with human ulcerative colitis (Paramsothy et al., 2017; Tahara et al., 2017), and been found to exacerbate inflammation in a murine colitis model through IL-17F and NF-κB signaling. Thus *F. nucleatum* may represent an inflammation-triggering commensal that is shared across species (Chen et al., 2020). While association of dysbiosis and intestinal inflammation are demonstrated in multiple equine studies, functional microbiota analyses, coupled with molecular analyses of equine cells, will allow a better understanding for how equine dysbiosis triggers inflammation.

## Bovine

The importance of microbiota composition in bovine health has long been recognized. The first compartment of cow’s forestomach, the rumen, holds 70–100 L of fluids composed of mutualistic flora and their metabolites, which are a major source of energy and nutrition of the cow. The cow’s diet is commonly formulated to maintain healthy rumen flora. Transfer of rumen fluid from a healthy cow to a cow with gastrointestinal disorders (transfaunation) has been practiced since the 18th century as a common therapeutic (DePeters and George, 2014). The microbiota of the cow intestine has received less attention than the rumen. Available data demonstrate a dominance of *Firmicutes*, suggesting there is continuous bacterial fermentation throughout the intestinal tract. Less abundant phyla in the cow intestines include *Bacteroidetes, Actinobacteria, Proteobacteria*, *Tenericutes*, and *Verrucomicrobia*. At the family/genus level, some of the representative taxa include *Ruminococcaceae, Christensenellaceae, Peptostreptococcaceae, Clostridiaceae, Bacteroides, Prevotella, Rikenellaceae, Butyrivibrio, Bifidobacterium, Campylobacter* and *Acinetobacter* (Figure 1; Mao et al., 2015; Fecteau et al., 2016; Li et al., 2020; Uchiyama et al., 2020). Functional analysis of the intestinal microbiota of cows has identified enriched pathways such as energy metabolism, replication and repair, amino acid metabolism, carbohydrate metabolism, and membrane transport. These indicate important roles of intestinal microbiota in nutrition and intestinal homeostasis.

Significant changes in microbiota composition in the intestine has been found in feedlot calves with hemorrhagic diarrhea with undefined etiology (Zeineldin et al., 2018). The calves with diarrhea had reduced *Ruminococcaceae, Flavobacteriaceae, Desulfohalobiaceae, Thermodesulfovibrionaceae*, and increased *Clostridia, Lachnospiraceae.* Some of the *Clostridia* in this data may have been pathogens, such as *C. perfringens.* Alpha-diversity of the microbiome was significantly decreased in the intestine of calves with diarrhea (Table 1; Zeineldin et al., 2018). IBD is not commonly diagnosed in cows, however, Johne’s disease, caused by *Mycobacterium avium* ssp. *paratuberculosis* (MAP) infection, shares many clinical and pathological features with Crohn’s disease (CD) of human (McNees et al., 2015). Because MAP infection has been controversially suspected as a cause of a subset of CD, a study was conducted to examine whether MAP infected, asymptomatic cows have dysbiosis (Fecteau et al., 2016). This work demonstrated dysbiosis in MAP-positive cows characterized by significantly decreased *Firmicutes* and *Bacteroidetes* and increased *Actinobacteria*. Because all animals were non-clinical in the study, the biological significance of this dysbiosis was unclear, though it was suggested as a potential predisposing factor for granulomatous enteritis and chronic enteropathy (Fecteau et al., 2016). Although the hind gut dysbiosis in bovine intestinal diseases are demonstrated in above studies, causal roles remain to be elucidated.

## Porcine

Because of the similarity of intestinal anatomy of pigs and humans, pigs are often employed as experimental models for human intestinal diseases. Thus, their intestinal microbiota has been studied under more well-controlled conditions, relative to other large animal species. *Firmicutes, Bacteroidetes* and *Proteobacteria* are the major phyla most commonly found in pig distal intestine. At lower taxonomic levels, genera *Clostridium*, *Blautia*, *Lactobacillus*, *Prevotella*, *Ruminococcus*, *Roseburia*, and *Subdoligranulum* represent some of the core commensal bacteria of the domestic pig (Allen et al., 2011; Holman et al., 2017; Han et al., 2018). While several pig models of experimentally induced intestinal inflammation have been described, naturally occurring IBD is not reported in domestic pigs. A longitudinal study examining microbiota of post-weaning pigs in association of development of diarrhea revealed significant dysbiosis prior to onset of diarrhea (Table 1; Dou et al., 2017). Multiple taxa including *Bacteroides, Ruminococcaceae, Oscillospira, Christensenellaceae, Prevotella*, and *Lachnospiraceae* were decreased; and *Enterococcus, Trueperella, Enterobacteriacea, Fusobacterium* and *Clostridium* were increased in the affected group prior to onset of diarrhea, compared to the healthy control pigs that never developed diarrhea. Unlike the other clinical studies in which duration or presence of dysbiosis prior to onset of intestinal inflammation is unknown, significant dysbiosis prior to the onset of diarrhea in this study suggests a potential causative role. Causes of the dysbiosis itself were not determined in this study. This study highlighted an advantage of natural porcine models in which a large number of similar age pigs can be examined at the same time in a controlled environment.

## Discussion and Future Directions

While most animal studies in this review did not employ statistical adjustment for confounding factors that could affect the intestinal flora, the findings support that dysbiosis plays a role in pathophysiology of intestinal inflammation across mammalian species. Aside from a subset of canine IBD and bovine MAP, the etiologies of IBD or non-infectious enteropathy in domestic animals are not attributed to a single pathogen or pathosymbiont. Rather, triggers may be associated with collective functional changes of microbiota and this effect on host cells, as proposed in human IBD (Ni et al., 2017). A recent systematic review of 48 human microbiome metagenomic studies did not identify any species that were universally associated with human IBD. However, multiple studies have identified common taxonomic perturbations in IBD, including increased *Escherichia* and decreased *Faecalibacterium*, which were also identified with IBD in dogs (Honneffer et al., 2014; Minamoto et al., 2015; Vázquez-Baeza et al., 2016; Pittayanon et al., 2020). A large human longitudinal study integrating metagenome, metatranscriptome and metabolome datasets did not identify taxa that commonly associate with IBD. Instead, functional profiling of microbiota, in combination with metabolite abundance, was more effective at separating IBD patients from controls (Lloyd-Price et al., 2019). While data examining functional alterations of microbiota in animal intestinal diseases are currently limited, a small number of dog and cat studies have described that the benzoate degradation pathway is increased in intestinal inflammation (Guard et al., 2015; Suchodolski et al., 2015). Interestingly, this pathway is similarly altered with human IBD (Gevers et al., 2014; Lewis et al., 2015; Tataru and David, 2020).

Among the domestic species, canine research is currently the most advanced with incorporation of functional profiling of microbiota and stratification of patient populations by treatment (Minamoto et al., 2015; Niina et al., 2019). The genetics of dogs due to inbreeding may be advantageous for further dissection of genome-microbiota-disease analyses, as fewer samples may be required to identify disease polymorphisms (Hayward et al., 2016). Human IBD studies geared towards precision medicine employ multiomics to study interactions of genetics, epigenetics, microbiota and metabolites (Imhann et al., 2018; Lloyd-Price et al., 2019; Ryan et al., 2020). This type of work has shown connections between host genotype and microbiota in human IBD (Putignani et al., 2019; Benech and Sokol, 2020). Specifically, IBD risk polymorphisms of NOD2, AGL16L1 and FUT2 genes were associated with compositional and functional changes in the microbiota (Frank et al., 2011; Rausch et al., 2011; Knights et al., 2014). Interestingly, canine NOD2 genes also associated with IBD in German shepherds (Kathrani et al., 2014). Other analyses of human metagenomics and metabolomics have found that outcomes of fecal transplantation reflected both the donor and recipient microbiota composition and function (Paramsothy et al., 2019). Rodent studies suggest microbiota composition may also alter the epigenetic status of mammalian cells (Krautkramer et al., 2016; Kelly et al., 2018; Ansari et al., 2020). In line with this, epithelial DNA methylation profiles were found to correlate with both dysbiosis and inflammation in IBD patients (Ryan et al., 2020). In addition, modeling metagenomic data to predict network dynamics are expected to guide microbiota manipulation. For example, use of the Lotka-Volterra model led to an algorithm to calculate fecal transplant efficiency and design personalized probiotic cocktails (Xiao et al., 2020). Thus, the next steps for taking advantage of comparative animal models and developing microbiota-based strategies for domestic species will require integration of multiomic host data with microbiome and metabolite analyses.

## Author Contributions

TA and SH-H planned and wrote the manuscript. Both authors contributed to the article and approved the submitted version.

## Conflict of Interest

The authors declare that the research was conducted in the absence of any commercial or financial relationships that could be construed as a potential conflict of interest.
